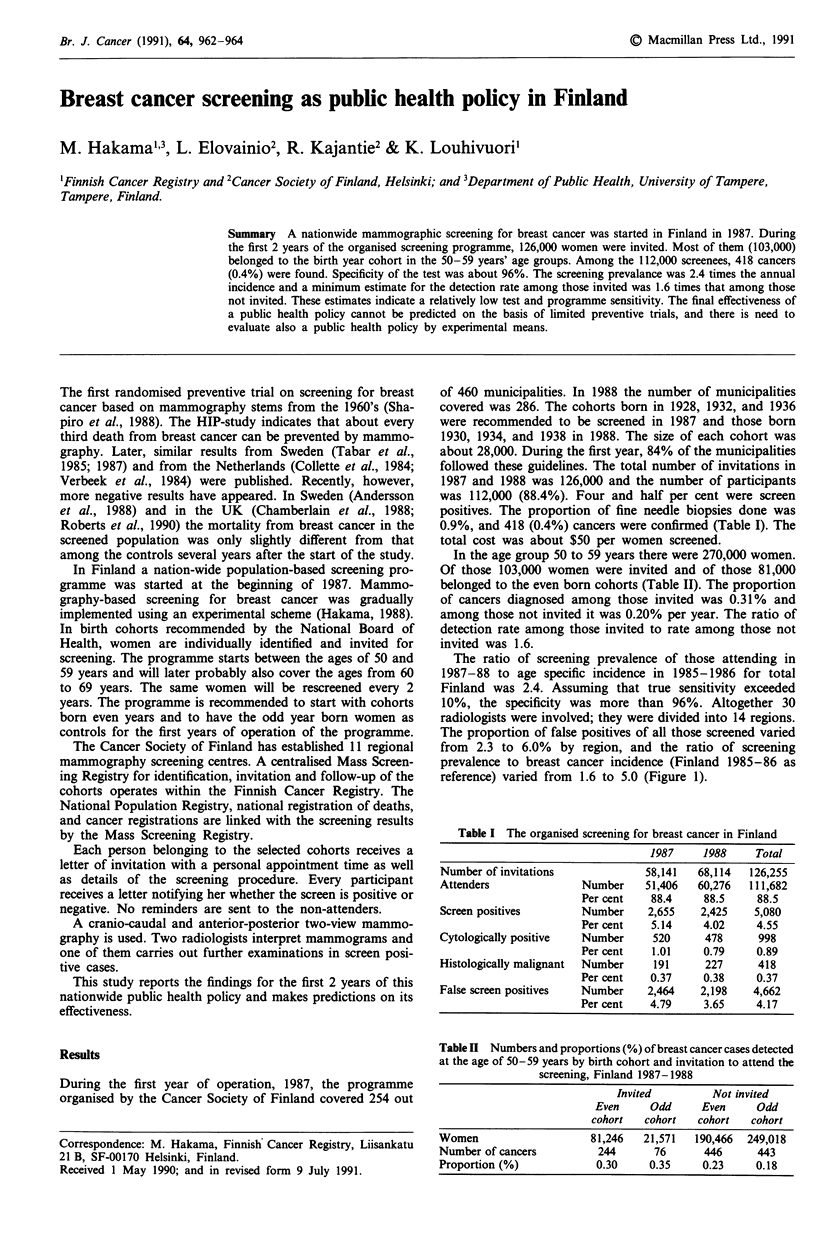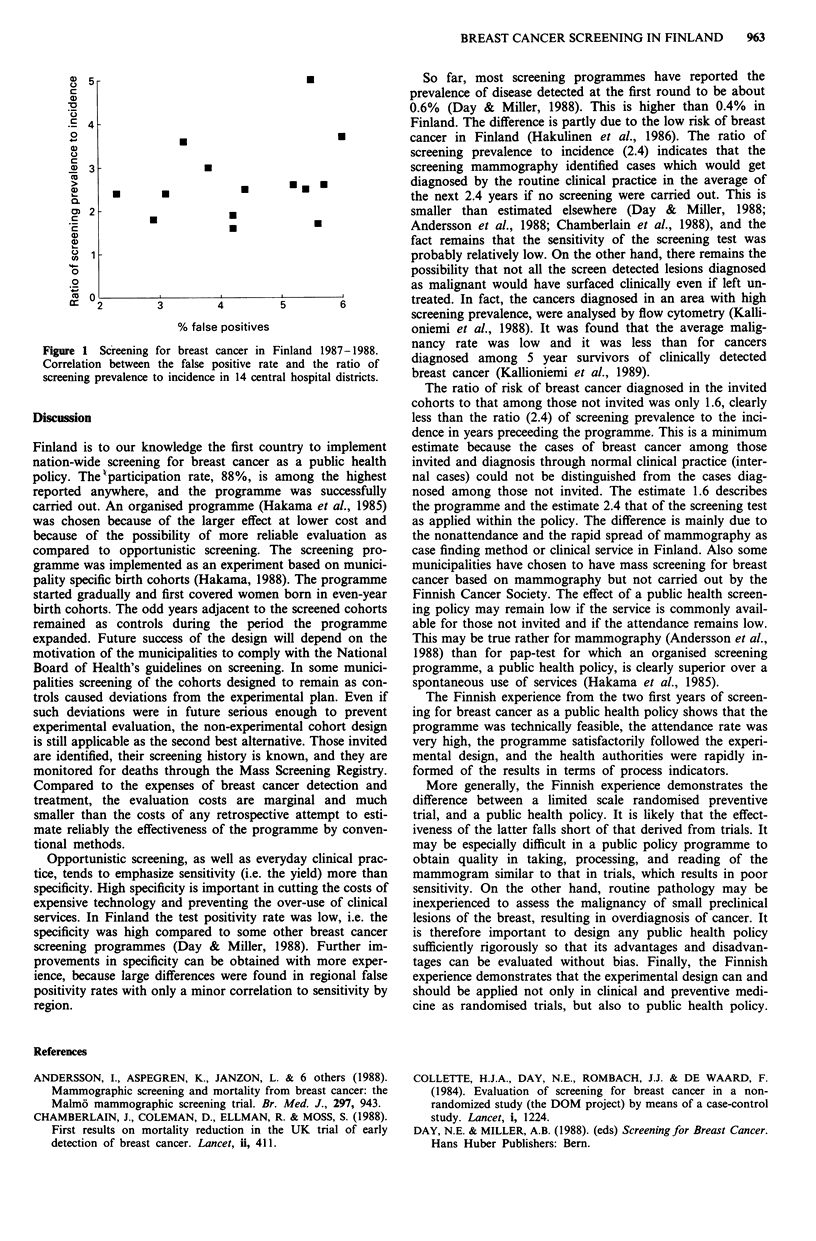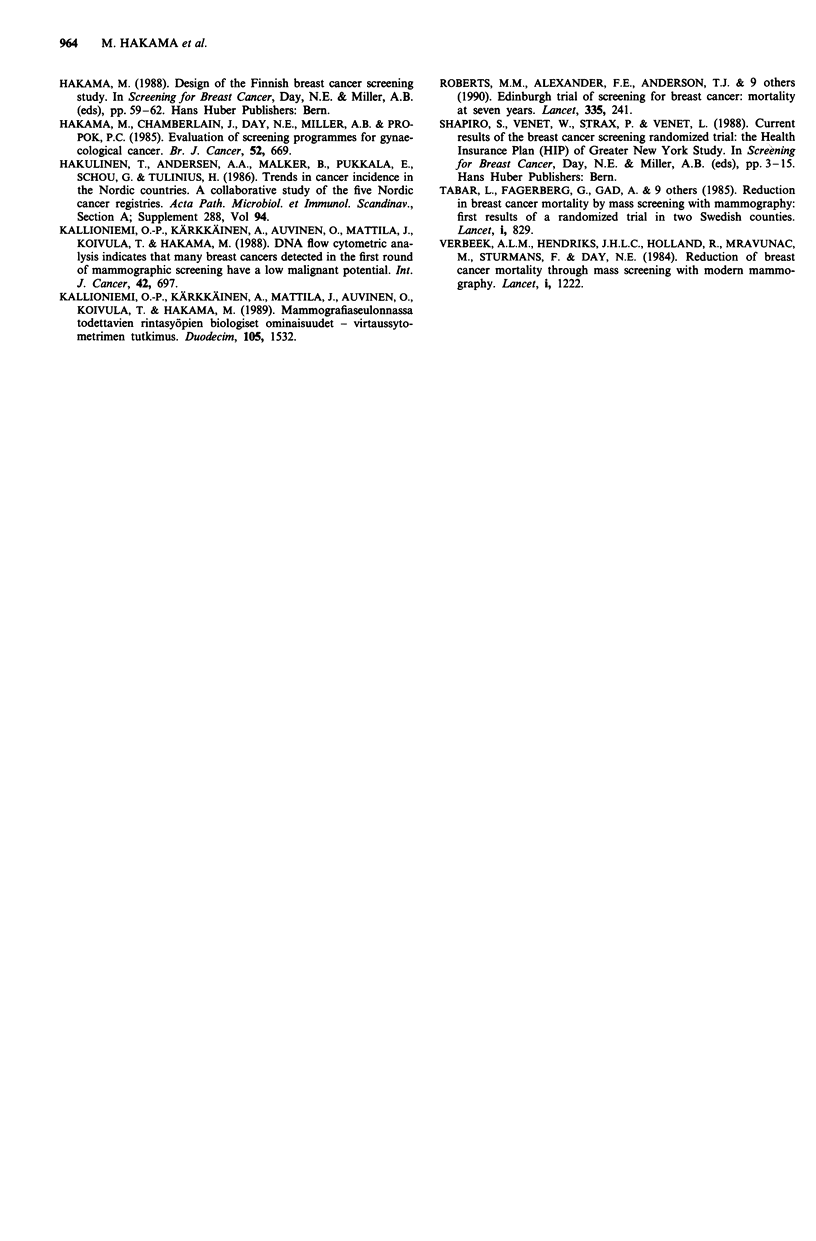# Breast cancer screening as public health policy in Finland.

**DOI:** 10.1038/bjc.1991.436

**Published:** 1991-11

**Authors:** M. Hakama, L. Elovainio, R. Kajantie, K. Louhivuori

**Affiliations:** Finnish Cancer Registry, Helsinki, Tampere, Finland.

## Abstract

A nationwide mammographic screening for breast cancer was started in Finland in 1987. During the first 2 years of the organised screening programme, 126,000 women were invited. Most of them (103,000) belonged to the birth year cohort in the 50-59 years' age groups. Among the 112,000 screenees, 418 cancers (0.4%) were found. Specificity of the test was about 96%. The screening prevalence was 2.4 times the annual incidence and a minimum estimate for the detection rate among those invited was 1.6 times that among those not invited. These estimates indicate a relatively low test and programme sensitivity. The final effectiveness of a public health policy cannot be predicted on the basis of limited preventive trials, and there is need to evaluate also a public health policy by experimental means.


Br. J. Cancer (1991), 64, 962-964 © Macmillan Press Ltd., 1991

### Breast cancer screening as public health policy in Finland

M. Hakama[1,3], L. Elovainio[2], R. Kajantie[2] & K. Louhivuori[1]

[1]*Finnish Cancer Registry and* [2]*Cancer Society of Finland, Helsinki; and* [3]*Department of Public Health, University of Tampere, Tampere, Finland.*

**Summary** A nationwide mammographic screening for breast cancer was started in Finland in 1987. During the first 2 years of the organised screening programme, 126,000 women were invited. Most of them (103,000) belonged to the birth year cohort in the 50-59 years' age groups. Among the 112,000 screenees, 418 cancers (0.4%) were found. Specificity of the test was about 96%. The screening prevalance was 2.4 times the annual incidence and a minimum estimate for the detection rate among those invited was 1.6 times that among those not invited. These estimates indicate a relatively low test and programme sensitivity. The final effectiveness of a public health policy cannot be predicted on the basis of limited preventive trials, and there is need to evaluate also a public health policy by experimental means.

The first randomised preventive trial on screening for breast cancer based on mammography stems from the 1960's (Shapiro *et al.*, 1988). The HIP-study indicates that about every third death from breast cancer can be prevented by mammography. Later, similar results from Sweden (Tabar *et al.*, 1985; 1987) and from the Netherlands (Collette *et al.*, 1984; Verbeek *et al.*, 1984) were published. Recently, however, more negative results have appeared. In Sweden (Andersson *et al.*, 1988) and in the UK (Chamberlain *et al.*, 1988; Roberts *et al.*, 1990) the mortality from breast cancer in the screened population was only slightly different from that among the controls several years after the start of the study.

In Finland a nation-wide population-based screening programme was started at the beginning of 1987. Mammography-based screening for breast cancer was gradually implemented using an experimental scheme (Hakama, 1988). In birth cohorts recommended by the National Board of Health, women are individually identified and invited for screening. The programme starts between the ages of 50 and 59 years and will later probably also cover the ages from 60 to 69 years. The same women will be rescreened every 2 years. The programme is recommended to start with cohorts born even years and to have the odd year born women as controls for the first years of operation of the programme.

The Cancer Society of Finland has established 11 regional mammography screening centres. A centralised Mass Screening Registry for identification, invitation and follow-up of the cohorts operates within the Finnish Cancer Registry. The National Population Registry, national registration of deaths, and cancer registrations are linked with the screening results by the Mass Screening Registry.

Each person belonging to the selected cohorts receives a letter of invitation with a personal appointment time as well as details of the screening procedure. Every participant receives a letter notifying her whether the screen is positive or negative. No reminders are sent to the non-attenders.

A cranio-caudal and anterior-posterior two-view mammography is used. Two radiologists interpret mammograms and one of them carries out further examinations in screen positive cases.

This study reports the findings for the first 2 years of this nationwide public health policy and makes predictions on its effectiveness.

### Results

During the first year of operation, 1987, the programme organised by the Cancer Society of Finland covered 254 out of 460 municipalities. In 1988 the number of municipalities covered was 286. The cohorts born in 1928, 1932, and 1936 were recommended to be screened in 1987 and those born 1930, 1934, and 1938 in 1988. The size of each cohort was about 28,000. During the first year, 84% of the municipalities followed these guidelines. The total number of invitations in 1987 and 1988 was 126,000 and the number of participants was 112,000 (88.4%). Four and half per cent were screen positives. The proportion of fine needle biopsies done was 0.9%, and 418 (0.4%) cancers were confirmed (Table I). The total cost was about $50 per women screened.

In the age group 50 to 59 years there were 270,000 women. Of those 103,000 women were invited and of those 81,000 belonged to the even born cohorts (Table II). The proportion of cancers diagnosed among those invited was 0.31% and among those not invited it was 0.20% per year. The ratio of detection rate among those invited to rate among those not invited was 1.6.

The ratio of screening prevalence of those attending in 1987-88 to age specific incidence in 1985-1986 for total Finland was 2.4. Assuming that true sensitivity exceeded 10%, the specificity was more than 96%. Altogether 30 radiologists were involved; they were divided into 14 regions. The proportion of false positives of all those screened varied from 2.3 to 6.0% by region, and the ratio of screening prevalence to breast cancer incidence (Finland 1985-86 as reference) varied from 1.6 to 5.0 (Figure 1).

**Table I** The organised screening for breast cancer in Finland

| | | *1987* | *1988* | *Total* |
|---|---|---|---|---|
| Number of invitations | | 58,141 | 68,114 | 126,255 |
| Attenders | Number | 51,406 | 60,276 | 111,682 |
| | Per cent | 88.4 | 88.5 | 88.5 |
| Screen positives | Number | 2,655 | 2,425 | 5,080 |
| | Per cent | 5.14 | 4.02 | 4.55 |
| Cytologically positive | Number | 520 | 478 | 998 |
| | Per cent | 1.01 | 0.79 | 0.89 |
| Histologically malignant | Number | 191 | 227 | 418 |
| | Per cent | 0.37 | 0.38 | 0.37 |
| False screen positives | Number | 2,464 | 2,198 | 4,662 |
| | Per cent | 4.79 | 3.65 | 4.17 |

**Table II** Numbers and proportions (%) of breast cancer cases detected at the age of 50-59 years by birth cohort and invitation to attend the screening, Finland 1987-1988

| | *Invited* | | *Not invited* | |
|---|---|---|---|---|
| | *Even cohort* | *Odd cohort* | *Even cohort* | *Odd cohort* |
| Women | 81,246 | 21,571 | 190,466 | 249,018 |
| Number of cancers | 244 | 76 | 446 | 443 |
| Proportion (%) | 0.30 | 0.35 | 0.23 | 0.18 |

Correspondence: M. Hakama, Finnish Cancer Registry, Liisankatu 21 B, SF-00170 Helsinki, Finland.
Received 1 May 1990; and in revised form 9 July 1991.

**Figure 1** Screening for breast cancer in Finland 1987–1988. Correlation between the false positive rate and the ratio of screening prevalence to incidence in 14 central hospital districts.

### Discussion

Finland is to our knowledge the first country to implement nation-wide screening for breast cancer as a public health policy. The participation rate, 88%, is among the highest reported anywhere, and the programme was successfully carried out. An organised programme (Hakama *et al.*, 1985) was chosen because of the larger effect at lower cost and because of the possibility of more reliable evaluation as compared to opportunistic screening. The screening programme was implemented as an experiment based on municipality specific birth cohorts (Hakama, 1988). The programme started gradually and first covered women born in even-year birth cohorts. The odd years adjacent to the screened cohorts remained as controls during the period the programme expanded. Future success of the design will depend on the motivation of the municipalities to comply with the National Board of Health's guidelines on screening. In some municipalities screening of the cohorts designed to remain as controls caused deviations from the experimental plan. Even if such deviations were in future serious enough to prevent experimental evaluation, the non-experimental cohort design is still applicable as the second best alternative. Those invited are identified, their screening history is known, and they are monitored for deaths through the Mass Screening Registry. Compared to the expenses of breast cancer detection and treatment, the evaluation costs are marginal and much smaller than the costs of any retrospective attempt to estimate reliably the effectiveness of the programme by conventional methods.

Opportunistic screening, as well as everyday clinical practice, tends to emphasize sensitivity (i.e. the yield) more than specificity. High specificity is important in cutting the costs of expensive technology and preventing the over-use of clinical services. In Finland the test positivity rate was low, i.e. the specificity was high compared to some other breast cancer screening programmes (Day & Miller, 1988). Further improvements in specificity can be obtained with more experience, because large differences were found in regional false positivity rates with only a minor correlation to sensitivity by region.

So far, most screening programmes have reported the prevalence of disease detected at the first round to be about 0.6% (Day & Miller, 1988). This is higher than 0.4% in Finland. The difference is partly due to the low risk of breast cancer in Finland (Hakulinen *et al.*, 1986). The ratio of screening prevalence to incidence (2.4) indicates that the screening mammography identified cases which would get diagnosed by the routine clinical practice in the average of the next 2.4 years if no screening were carried out. This is smaller than estimated elsewhere (Day & Miller, 1988; Andersson *et al.*, 1988; Chamberlain *et al.*, 1988), and the fact remains that the sensitivity of the screening test was probably relatively low. On the other hand, there remains the possibility that not all the screen detected lesions diagnosed as malignant would have surfaced clinically even if left untreated. In fact, the cancers diagnosed in an area with high screening prevalence, were analysed by flow cytometry (Kallioniemi *et al.*, 1988). It was found that the average malignancy rate was low and it was less than for cancers diagnosed among 5 year survivors of clinically detected breast cancer (Kallioniemi *et al.*, 1989).

The ratio of risk of breast cancer diagnosed in the invited cohorts to that among those not invited was only 1.6, clearly less than the ratio (2.4) of screening prevalence to the incidence in years preceeding the programme. This is a minimum estimate because the cases of breast cancer among those invited and diagnosis through normal clinical practice (internal cases) could not be distinguished from the cases diagnosed among those not invited. The estimate 1.6 describes the programme and the estimate 2.4 that of the screening test as applied within the policy. The difference is mainly due to the nonattendance and the rapid spread of mammography as case finding method or clinical service in Finland. Also some municipalities have chosen to have mass screening for breast cancer based on mammography but not carried out by the Finnish Cancer Society. The effect of a public health screening policy may remain low if the service is commonly available for those not invited and if the attendance remains low. This may be true rather for mammography (Andersson *et al.*, 1988) than for pap-test for which an organised screening programme, a public health policy, is clearly superior over a spontaneous use of services (Hakama *et al.*, 1985).

The Finnish experience from the two first years of screening for breast cancer as a public health policy shows that the programme was technically feasible, the attendance rate was very high, the programme satisfactorily followed the experimental design, and the health authorities were rapidly informed of the results in terms of process indicators.

More generally, the Finnish experience demonstrates the difference between a limited scale randomised preventive trial, and a public health policy. It is likely that the effectiveness of the latter falls short of that derived from trials. It may be especially difficult in a public policy programme to obtain quality in taking, processing, and reading of the mammogram similar to that in trials, which results in poor sensitivity. On the other hand, routine pathology may be inexperienced to assess the malignancy of small preclinical lesions of the breast, resulting in overdiagnosis of cancer. It is therefore important to design any public health policy sufficiently rigorously so that its advantages and disadvantages can be evaluated without bias. Finally, the Finnish experience demonstrates that the experimental design can and should be applied not only in clinical and preventive medicine as randomised trials, but also to public health policy.

### References

ANDERSSON, I., ASPEGREN, K., JANZON, L. & 6 others (1988). Mammographic screening and mortality from breast cancer: the Malmö mammographic screening trial. *Br. Med. J.*, **297**, 943.

CHAMBERLAIN, J., COLEMAN, D., ELLMAN, R. & MOSS, S. (1988). First results on mortality reduction in the UK trial of early detection of breast cancer. *Lancet*, **ii**, 411.

COLLETTE, H.J.A., DAY, N.E., ROMBACH, J.J. & DE WAARD, F. (1984). Evaluation of screening for breast cancer in a non-randomized study (the DOM project) by means of a case-control study. *Lancet*, **i**, 1224.

DAY, N.E. & MILLER, A.B. (1988). (eds) *Screening for Breast Cancer*. Hans Huber Publishers: Bern.

HAKAMA, M. (1988). Design of the Finnish breast cancer screening study. In *Screening for Breast Cancer*, Day, N.E. & Miller, A.B. (eds), pp. 59-62. Hans Huber Publishers: Bern.

HAKAMA, M., CHAMBERLAIN, J., DAY, N.E., MILLER, A.B. & PROPOK, P.C. (1985). Evaluation of screening programmes for gynaecological cancer. *Br. J. Cancer*, **52**, 669.

HAKULINEN, T., ANDERSEN, A.A., MALKER, B., PUKKALA, E., SCHOU, G. & TULINIUS, H. (1986). Trends in cancer incidence in the Nordic countries. A collaborative study of the five Nordic cancer registries. *Acta Path. Microbiol. et Immunol. Scandinav.*, Section A; Supplement 288, Vol **94**.

KALLIONIEMI, O.-P., KÄRKKÄINEN, A., AUVINEN, O., MATTILA, J., KOIVULA, T. & HAKAMA, M. (1988). DNA flow cytometric analysis indicates that many breast cancers detected in the first round of mammographic screening have a low malignant potential. *Int. J. Cancer*, **42**, 697.

KALLIONIEMI, O.-P., KÄRKKÄINEN, A., MATTILA, J., AUVINEN, O., KOIVULA, T. & HAKAMA, M. (1989). Mammografiaseulonnassa todettavien rintasyöpien biologiset ominaisuudet – virtaussytometrimen tutkimus. *Duodecim*, **105**, 1532.

ROBERTS, M.M., ALEXANDER, F.E., ANDERSON, T.J. & 9 others (1990). Edinburgh trial of screening for breast cancer: mortality at seven years. *Lancet*, **335**, 241.

SHAPIRO, S., VENET, W., STRAX, P. & VENET, L. (1988). Current results of the breast cancer screening randomized trial: the Health Insurance Plan (HIP) of Greater New York Study. In *Screening for Breast Cancer*, Day, N.E. & Miller, A.B. (eds), pp. 3-15. Hans Huber Publishers: Bern.

TABAR, L., FAGERBERG, G., GAD, A. & 9 others (1985). Reduction in breast cancer mortality by mass screening with mammography: first results of a randomized trial in two Swedish counties. *Lancet*, **i**, 829.

VERBEEK, A.L.M., HENDRIKS, J.H.L.C., HOLLAND, R., MRAVUNAC, M., STURMANS, F. & DAY, N.E. (1984). Reduction of breast cancer mortality through mass screening with modern mammography. *Lancet*, **i**, 1222.